# Reporting COVID-19 A Conversation with Jon Cohen of Science

**DOI:** 10.4269/ajtmh.20-1034

**Published:** 2020-08-25

**Authors:** Claire Panosian Dunavan

**Affiliations:** University of California; Los Angeles, California; E-mail: cpanosian@mednet.ucla.edu

On July 31, 2020, Science magazine published long-awaited responses from Shi Zhengli of the Wuhan Institute of Virology. “China’s ‘Bat Woman’ denies responsibility for the pandemic, demands apology from Trump” read the article’s subtitle, followed by a familiar byline. Jon Cohen’s usual beat includes epidemics, immunology, global health, and especially HIV/AIDS. This year, by contrast, 95% of his work has dealt with COVID-19.

Cohen is ideally suited to cover the pandemic, and not only because he possesses a wealth of experience reporting infectious diseases. There’s also his Rolodex. To date, he’s visited more than 50 countries for Science and can easily tap sources from Anthony Fauci to leading researchers in China.

Writing from California, where the senior correspondent established the West Coast bureau for Science, Cohen has also published in *The New Yorker*, *Atlantic Monthly*, *The New York Times Magazine*, BuzzFeed, *Smithsonian*, *Slate*, and *Surfer*, and authored four books for lay readers. His print journalism has been honored by the U.S. National Academy of Sciences, the National Association of Science Writers, the American Society of Microbiology, the American Society of Tropical Medicine and Hygiene, the Pan American Health Organization, and the Council for the Advancement of Science Writing. In 2017, Cohen also won a national Emmy for helping to develop and appearing in a six-part series about AIDS on the *PBS NewsHour*.

How did Jon Cohen earn his stripes? “I have a Jewish mother,” he recently told me, “I was basically programmed to be a doctor.” But in 1980, a change of heart prompted a different plan. During his final year at the University of California, San Diego, he completed a self-designed major by 1) interviewing Jonas Salk at length, then writing a 10,000 word paper about polio vaccines that Salk critiqued; 2) authoring two children’s books about science; and 3) translating a scientific research article into “plain English” to show that it was indeed possible to impart technical content to lay readers.

From there, his path took twists and turns. But a key turning point came when Cohen landed a job in Washington, DC. This post launched his life in journalism and, curiously enough, reawakened his early interest in vaccines, which is where our conversation began.

## INTERVIEW WITH JON COHEN^†^

One of the interesting things to me about the COVID-19 pandemic is how much time I have spent looking at the past. Not long ago, I spent a day with my 90-year-old mother in Los Angeles. We went to Topanga Canyon, visited the beach, and went through boxes of stuff that I had left in her house. In one of those boxes, I found a report I wrote in seventh or eighth grade that was based on a beautiful book about Jonas Salk and the search for a polio vaccine.^‡^

In the book, Jonas Salk receives advice from an elderly scientist who tells him: “Do something that makes your heart leap.” Well, in high school and college, I was always writing. And, every time I would publish something, my heart would leap, whereas every time I got an A on an organic chemistry test, I would think: “Okay, I can do this.” I also loved writers. They were my people. Even now, when I get in a roomful of writers, I just light up.

##  You credit a free, weekly newspaper in Washington, DC, for honing your craft. Please share how working for the *Washington City Paper* reconnected you with Jonas Salk, an event which ultimately led to your first job at Science.

At that paper, I covered the city, Marion Barry, everything but science. But in 1987, we had a crisis. A story didn’t show up on time. So I said to the editor: “Look, I’ve got this old story in my file that I wrote about Jonas Salk… let me see if he’ll free me from my commitment to not run it.” And I called Jonas Salk, and he took my call and said: “Don’t run that story. There’s a better story. My son is testifying in Baltimore about a man who died from polio, which he contracted from his baby daughter after she received the live, oral polio vaccine.”

It was the first piece of science writing I ever published and, to this day, it’s the story that I’m probably most proud of. The man and his wife met on the 19th, he developed polio on the 19th, he died on the 19th. So the open was: “19 used to be Debbie’s lucky number,” and it got picked up by free weeklies all over the country and got a tremendous amount of attention.

Then in 1989, I read a *Washington Post* story about Jonas Salk collaborating with someone at the NIH on an AIDS vaccine. And I had done a few AIDS stories, but it wasn’t my beat. I went to meet that scientist—a guy named Joe Gibbs who once worked with Carleton Gajdusek, the Nobel Prize winner. Then, I came back and talked to my editor. “What’s the story?” he asked. And I said: “I don’t know if there’s a story.” And I told him what the guy said. He said: “Jon, you’re an idiot. The story is the same as the polio vaccine story. This is Jonas Salk, once again going against the trend, pushing for a killed virus vaccine when everyone else is making genetically engineered recombinant proteins of gp 160 or gp 120.”

So in 1989, I wrote what I think is the first critical analysis ever published of the AIDS vaccine search that asked the question: “Why is everybody addicted to the sexy, new technology when the old-fashioned technology appears to be working better in monkeys and chimpanzees?” And an agent called me and said: “Do you have any interest in writing a book?” So I came up with possibly the stupidest idea of all time for a book: 1 year in the search for an AIDS vaccine. Because I was certain Jonas Salk was going to be right and have a vaccine.

Anyway, I got a book contract and about 6 months into it, I was interviewing Robert Gallo^§^ and he said, in a flippant way, “…this year, that is so important to you.” And I couldn’t get that out of my head because I realized it was a conceit of mine as a writer; in the scientific world, a year in the search for a vaccine was ridiculous. So I wrote about half [of the book], and my wife Shannon got really tired of my not making money. Plus, we had a baby. So I picked up the phone and I called Science magazine, which I barely read. It was a Friday afternoon, and a top editor happened to answer the call. I pitched an idea that I had learned while reporting this book. And he said: “Yeah, do the story.” I did it, it ran in September 1990, and then he asked if I had more ideas. I had a long list.

The book,^‖^ which finally became a polemic about the wayward search for an AIDS vaccine, catalogs how disorganized the field was and how promising leads languished. It basically made an argument for a Manhattan Project for an AIDS vaccine. And here we are facing the same issues with COVID-19. This time around, maybe 1 year in the search isn’t a bad idea for a book.

### We’ll get to COVID soon, but for starters, please talk a bit more about science writing and qualities you value in your sources.

I’ve always had a very broad definition of what science writing is. I cover anything I can convince my editors has a science angle. I also see myself as a professional student, someone who never graduates. My sources are my professors. They’re people, who, ideally, know more than I do and are willing to suffer through my learning curve.

Yes, at this point, I’m bilingual. I speak English and science. I’m also bilingual in niches like immunology and virology, so with many sources I can cut to the chase really quickly. But on top of that, I go into the field and I see things that many of them don’t often see. I go into villages that are taking part in a clinical study, for example. Or I go into a pharmaceutical company and tour their bioreactor plant. I end up becoming what I like to think of as a two-way street for my sources, where I’m not just taking information, I’m sharing information. Which is ultimately my job, right?

Because of the way I was raised, I’m also not shy about contacting strangers. I was raised in a very garrulous household where everybody talked at the same time. That’s why, I think, I’ve been able to succeed as a journalist. I truly enjoy meeting people. I talk a lot, but I listen more.

### Let’s move on to HIV, which has been a major launchpad for your coverage of COVID. Looking back, can you share some highlights?

For me, what was really fortunate is that AIDS is a syndrome that touches on many different diseases. I had to learn all these different opportunistic bugs, not just HIV. HIV is syndemic with tuberculosis, so TB became part of my beat, part of my passion. And frankly, TB is more frightening to me personally. If I go into a hospital where everyone is dying from AIDS, I’m at zero risk of getting infected with HIV. But when I walk into Durban’s hospital for extensively drug-resistant tuberculosis and I’m interviewing patients who all have XDR TB, I’m at high risk. I don’t want to become infected.

HIV has put me into a situation again and again where I have to be careful and knowledgeable. I’m also meeting with clinicians and healthcare workers, and with people living with different viruses and bacteria who I need to approach with respect, compassion, and humility.

Because it’s my job, when I go to Kinshasa, I go to a brothel. And when I’m in Manipur, in India, I go to a room where people are shooting heroin. HIV has introduced me to transgender and gay communities. I love to be the anti-tourist, to meet people on the ground, and to watch researchers do research, much of which is identical to journalism. You’re collecting enormous amounts of information and you’re going to distill it with a story. We don’t typically speak about scientific papers as being stories, but they are.

### This brings us to your recent face-to-face encounter with COVID-19.

Yes, a few weeks ago, I was invited to the busiest COVID-19 ICU in San Diego. It was such a relief to me to go back into the field because I’ve been locked up in my office for 6 months and unable to do what I love most, which is to see people doing their jobs.

When I finally got into the ICU and I PPE’ed up, I realized everyone was on Versed and Fentanyl because they were being ventilated. I couldn’t interview patients, but watching the doctors and nurses do their work and learning that there were chaplains in ICUs was really eye-opening to me. Great storytelling is not about “telling,” it’s showing what you’ve seen, and so much of the reporting I’ve been forced to do has been telling because I’m mainly on Zoom calls.

### Exactly. For the last 6 months, you’ve been chained to your desk. How’s that been for you?

I’ve been a productive journalist, anyway. I have never produced more words since diving into this on January 8, and I’ve never been more confused about what day of the week it is and even what time it is during the day. I guess it’s like being drunk. I lose a sense of time and place because I’m so involved with what I’m doing. But when I’ve stepped back from it, I’ve felt useful. I feel like I’m connecting the dots in my career and that I’m helping to clarify critical things.

I still find original angles—almost every day I wake up to something. So yeah, I get burned out, but I also get the fire in my belly. It’s pretty easy to get me going with a story if I know something other people don’t know that is both significant and interesting. I live for that.

### When did you know that this would be a global pandemic?

Oh, I knew by the third week in January that we and the world were in trouble. Jeremy Farrar^¶^ was the first person I quoted who publicly said this wouldn’t end in China, that it would likely go global.

### What insights can you share about key sources, both here in the United States and in China?

Well, there’s something profound in my life about COVID-19. The people at the front—Tony Fauci, Robert Redfield, and Debbie Birx—many of them came out of the HIV world. I recently interviewed epidemiologist Shao Yiming^#^ because he was my official sponsor when I went to China in 2004. People who know me through the HIV world also suggested that I interview George Gao, the head of the Chinese CDC, and encouraged him to speak with me. In addition, many of the COVID-19 vaccine efforts are building directly out of HIV vaccine efforts. And the monoclonal antibody effort is building, in part, off a current study of monoclonals for treatment and prevention of HIV. So there’s a strong link between many of my sources and my experience covering HIV. Plus I’ve worked all over China and met with researchers and laboratories. I know how to use WeChat, and I can speak to anybody anywhere with Google Translate.

I will, in the next few days, have an interview published with Shi Zenghli, the researcher in Wuhan who’s sometimes called “Bat Woman” in China. She’s been very reluctant to speak to the media. Over the last 2 months, I sent her a very long list of questions and she has answered them. I think it will be the first time she’s publicly addressed many key theories around the laboratory potentially being a source of the virus, which she denies, but she’s addressing specifics in my interviews with her.

### It sounds like you’ve become something of a diplomat.

Well, I’m a neutral party. I am Switzerland. I don’t represent the Trump administration, certainly. The flag I fly is the flag of science, which is an international flag. I’ve interviewed a doctor in Italy who got COVID in northern Italy and donated his own convalescent plasma to advance the development of monoclonal antibodies. And I’ve interviewed people who run the biggest vaccine company in India and people in Brazil. One of the advantages of COVID-19 is that everyone’s around. Nobody can fall back by saying “Oh, I’m traveling.”

I should add, however, that China is not open about letting scientists speak to journalists. It’s always a negotiation. Every communication feels strained. They want questions in writing, and they want to answer in writing. They certainly are circulating the questions and answers for approval. China does not believe in freedom of the press, and it doesn’t believe in open scientific communication either. Let’s not delude ourselves into thinking that they are playing by the same rules. They are not. The most prominent individual scientists, however, have lived in the United States or Europe or Australia where information moves freely. So, especially people who are working on infectious diseases are dancing a dance to not get in trouble with party officials while remaining respectful international scientists.

##  Tell me more about your interviews with Tony Fauci.

I still speak with Tony with some regularity. Tony was famous before this, but he’s now a household name. In the world of science, lots of people knew who Tony Fauci was, and in the world of HIV/AIDS, the community knew who he was. But now that he’s become an icon and a representative of speaking truth to power, it’s a very fine dance. As he made clear in my blunt interview with him, he struggles with it, with how best to influence the White House without being ignored. This whole idea of “Fire Fauci” is nonsense. Trump cannot fire Fauci. He could kick him off the coronavirus task force, but he doesn’t have the authority to fire him. I also find Tony to be as politically savvy as any scientist I’ve ever met. I think he understands that Trump benefits from tension in their relationship. [Even] when Trump bad-mouths Tony, or has his people take shots at him, he still wants him around.

You also have to see Tony through the lens of his upbringing. He’s a Jesuit-trained person from New York and Trump is from New York. They speak a New York dialogue with each other. And as a Jesuit-trained person, Tony is used to absorbing insults, moving with a goal in mind that is, if you will, a higher purpose.

### What about CDC’s current role?

Robert Redfield has never had the public presence that Tony has. He tends to shy away from conflict and doesn’t want to rock boats. The CDC has been sidelined and has not been holding press conferences since Nancy Messonnier upset the White House on February 25 when she said this was going to be severe. She was right, and she was punished for it. Michael Kinsley, founding editor of *Slate* magazine, said that a gaffe in Washington for a politician is telling the truth. Messonnier was punished for telling the truth. Since then, the CDC has basically walked away from its role of being the lead agency informing the public about what to do. It’s allowed the White House to weigh in and change CDC’s scientific recommendations in ways that are not scientific. All of that chips away at the CDC’s reputation.

During the H1N1 pandemic, my best sources were frontline responders at CDC. They were great. Now, are you kidding? They’re not allowed to speak.

### Are you concerned that the national data bank is now at the Department of Health and Human Services as opposed to the CDC?

Personally, I don’t know enough to be concerned, but people I respect, including four former CDC directors, are concerned. That should make all of us concerned.

### What can you say about serial COVID infections.

We all have to grapple with living with uncertainty. That’s what COVID has done that’s most unsettling. We can’t answer these fundamental questions about whether our kids can go to school, or when we will return to this normal or the new normal. The idea that people can have very short-lived immunity, I think, remains to be seen. Certainly, there will be a bell-shaped curve, and there will be some people who can be reinfected with the same or a slight variant in short order. I imagine history will repeat itself and most people will have some sort of durable immunity that lasts a year or years.

### Would you participate in a clinical trial of a COVID vaccine?

Would I do it if I didn’t cover them? Yeah, I wouldn’t have any reservation about taking part in a trial. I don’t see any candidate vaccines that look particularly dangerous. It’s always possible that an experimental medicine could have serious side effects, but I haven’t seen evidence of anything in animal models or human trials that raise red flags for me. So yes, I would. I don’t fear them and I’m altruistic enough that I would volunteer.

### What’s your take on human challenge trials that require volunteers to be infected with SARS-CoV-2?

They’ve gained momentum. There are two central questions. One is: can they be done ethically. The other is: can they really provide information before efficacy trials based on natural exposure and infection with wild-type virus. The time line for conducting challenge trials is dependent on creating a virus that’s grown under good manufacturing practice (GMP) and then is put through a series of dose escalation studies in humans. All of that takes time. I just did a story about this the other day. There are two laboratories now that say they are going to make GMP-quality virus by September. Then you can start those dose escalation studies. The WHO has written a guideline about how to do them.

The ethical questions are going to hinge in part on whether there’s a rescue treatment. For malaria vaccine trials, where we have a challenge model, if somebody gets malaria, you can treat them very quickly. The same is true of cholera and challenge studies. With influenza, even, they use Tamiflu, or they have it at the ready, and they have antibiotics for secondary infections.

There’s another curious question. If you do the study without a rescue drug and you do it in 18–25 year olds who are unlikely to develop severe disease, even if everything goes well: what does the data mean for 80 year olds?

I don’t think this is 1955 with polio. I don’t think we’re going to arrive at a vaccine and church bells are going to ring and people are going to dance in the streets. I think we’re going to have a vaccine that shows some degree of efficacy and we’ll say right off the bat, we can do better than this. Then, there will be a second vaccine and the third vaccine and the fourth one, and there’ll be one for the elderly and there’ll be one for the kids and there’ll be one for people with diabetes. We’ll start to see COVID-19 vaccines as a suite of different vaccines.

### What is it like to be living through a time when vaccinology has moved so fast?

A lot of ideas that are becoming popular are ideas I’ve been writing and thinking about for a very long time. Last year, I did a story about the durability of vaccine immunity. In October, before COVID hit, I started working on a story about seasonality of disease. These are the topics of vaccinology that have fascinated me since I was 14 years old. What makes a good vaccine? At the core of it is memory.

I mentioned to you going through this box with my mother and finding the polio vaccine report and the memories it stirred. That’s what vaccines are doing. They’re trying to artificially create memory and recollection. That’s what’s so mesmerizing about vaccines.

### Let’s hope recollection means we don’t repeat today’s mistakes in the future.

Well, exactly. What we should be doing with this is building a pan-coronavirus vaccine and a pan-influenza vaccine. We should be pushing for these dreams of universal vaccination against categories of pathogens with a fervor that remembers what this cost us.

## Footnotes

† This interview was conducted by phone on July 22, 2020 and was later edited for clarity and length.

‡ Richard Carter’s *Breakthrough: The Saga of Jonas Salk* was published in 1966 by Trident Press.

§ Dr. Robert Gallo, who worked at the National Cancer Institute at NIH for 30 years, is internationally recognized as the codiscoverer of HIV as the cause of AIDS.

‖ Jon Cohen’s first book, *Shots in the Dark—The Wayward Search for an AIDS Vaccine*, was published in 2001 by W. W. Norton & Company.

¶ Jeremy Farrar, the director of the Wellcome Trust, was cited as follows in Cohen’s story of January 14, 2020: “Limited as the outbreak appears to date, Farrar and others still worry that travel of hundreds of millions of people for the Lunar New Year celebration on January 25 could spread the virus... ‘With people, food and animals move,’ says Farrar, who suspects that this outbreak ‘is not going away anytime soon.”

# Shao Yiming is currently chief expert on AIDS at the Chinese Center for Disease Control and Prevention.
